# Social Media and Medical Education in the Context of the COVID-19 Pandemic: Scoping Review

**DOI:** 10.2196/25892

**Published:** 2021-04-12

**Authors:** Marc Katz, Neilanjan Nandi

**Affiliations:** 1 Division of Cardiology Department of Medicine St. Luke's University Hospital Bethlehem, PA United States; 2 Division of Gastroenterology and Hepatology Department of Medicine University of Pennsylvania Philadelphia, PA United States

**Keywords:** social media, medical education, COVID-19, medical student, review, doctor, communication, online learning, e-learning, online education, delivery, dissemination

## Abstract

**Background:**

The COVID-19 pandemic has brought virtual web-based learning to the forefront of medical education as training programs adapt to physical distancing challenges while maintaining the rigorous standards of medical training. Social media has unique and partially untapped potential to supplement formal medical education.

**Objective:**

The aim of this review is to provide a summary of the incentives, applications, challenges, and pitfalls of social media–based medical education for both trainees and educators.

**Methods:**

We performed a literature review via PubMed of medical research involving social media platforms, including Facebook, Twitter, Instagram, YouTube, WhatsApp, and podcasts. Papers were reviewed for inclusion based on the integrity and power of the study.

**Results:**

The unique characteristics of social media platforms such as Facebook, Twitter, Instagram, YouTube, WhatsApp, and podcasts endow them with unique communication capabilities that serve different educational purposes in both formal and informal education settings. However, contemporary medical education curricula lack widespread guidance on meaningful use, application, and deployment of social media in medical education.

**Conclusions:**

Clinicians and institutions must evolve to embrace the use of social media platforms for medical education. Health care professionals can approach social media engagement in the same ethical manner that they would with patients in person; however, health care institutions ultimately must enable their health care professionals to achieve this by enacting realistic social media policies. Institutions should appoint clinicians with strong social media experience to leadership roles to spearhead these generational and cultural changes. Further studies are needed to better understand how health care professionals can most effectively use social media platforms as educational tools. Ultimately, social media is here to stay, influencing lay public knowledge and trainee knowledge. Clinicians and institutions must embrace this complementary modality of trainee education and champion social media as a novel distribution platform that can also help propagate truth in a time of misinformation, such as the COVID-19 pandemic.

## Introduction

Social media has become an integral vehicle for the delivery and dissemination of health care education. Although social media use has become ubiquitous among patients, health care practitioners have shown variable enthusiasm with regard to adoption and engagement within the social media realm. The COVID-19 pandemic has brought virtual web-based learning to the forefront of medical education as training programs adapt to physical distancing challenges while maintaining the rigorous standards of medical training. Social media offers unique and partially untapped potential to supplement formal medical education. Indeed, social media has also provided clinicians who must practice social distancing for public safety with an opportunity and virtual space for educational discourse, community, camaraderie, and support. Notably, contemporary curricula on the application, deployment, and professional etiquette of social media are lacking. In this review, we provide a summary of the incentives and applications of social media–based medical education for both trainees and educators. Likewise, we highlight the challenges and pitfalls of social media–based medical education.

## Methods

We performed a literature review by searching PubMed for medical research studies involving social media platforms, including Facebook, Twitter, Instagram, WhatsApp, and podcasts. Papers were reviewed for inclusion based on the integrity and power of the study.

## Results

### Social Media: History, Evolution, and Use Prevalence

A social media platform is characterized as a web-based application that facilitates interactive creation and sharing of information and ideas through virtual communities. Facebook, Twitter, Instagram, YouTube, WhatsApp, and various podcast-hosting applications are among the most popular and established electronic communication tools and social media platforms. Each platform has its own individual smartphone mobile app with unique user interfaces. These individual platforms have variable degrees of flexibility and limitation on how content is posted. Twitter permits a total of 280 characters in a single tweet, whereas other platforms may be far more generous; for example, Facebook permits up to 63,206 characters in a single post. Images and videos are permitted on all platforms; however, the number of images and the permitted video length may differ between these platforms. Instagram is intentionally built to share images and short videos. YouTube is strictly built for videos and does not restrict video length. WhatsApp provides secure, encrypted messaging and sharing of audiovisual material capabilities within closed groups; however, it is restricted to mobile devices and does not have a traditional desktop, web-based user interface. These platform-specific parameters enable each social media platform to be used uniquely for different types of educational learning.

Critical to the global adoption of social media platforms is the parallel and complementary development of high-speed internet and smart devices, which laid the groundwork for their creation and global adoption. The ability to capture and share high-quality audiovisual media evolved from basic email and text messaging to dissemination of such media via social networks, with social network access transitioning from a computer interface to a smartphone interface. The prevalence of smartphone technology is undoubtedly widespread in the United States, with the estimated number of Americans who owned a smartphone rising from 56% in 2013 to 77% in 2017 [1]. Similarly, smart tablet use in America rose from 3% in 2010 to 51% in 2016 [2]. Social media platforms have similarly experienced widespread multigenerational adoption. In 2014, the percentage of Americans who reported using smartphones to access social media was 55% in those aged more than 50 years, 77% in Americans aged 30-49 years, and 91% in Americans aged 18-29 years [3]. The percentage of American adults who used at least one social media platform rose from 5% in 2005 to 72% in 2019. Additionally, in 2019, an estimated 75% of Facebook users, 63% of Instagram users, and 42% of Twitter users reported accessing each social media platform, respectively, on a daily basis [3]. Hence, the critical focus on the word “media” in social media bears much weight and recognition in considering the ramifications of how social media has changed society over the last 15 years as social media applications have become a part of daily life.

### Physician Engagement on Social Media Before and During the COVID-19 Pandemic

Prior generations of physicians were apprehensive about engaging on social media out of concern about patient privacy, liability, lack of time, compensation, and familiarity with the technology; however, times are changing [4,5]. In a 2011 survey of 4033 clinicians, it was found that 90% of clinicians used at least one social media site for personal use and that 65% of clinicians already used at least one social media platform for professional purposes [6]. Many physicians use social media to find and share health information, communicate with colleagues and trainees, advertise their clinical practices, engage in health advocacy, impact health policy decisions, exchange developments in their fields, and publicize their research [7-12]. Over 140 uses for Twitter alone have been reported in health care [8]. Beyond social networking, clinicians have historically used social media platforms to directly engage and educate professional peers, house staff trainees, and patients. 

The advent of COVID-19 further catalyzed the adoption of social media platforms such as Twitter to more rapidly disseminate and spread information about an unknown and contagious disease directly to frontline reporters as new information unfolded. This was critical in many instances, such as providing guidance on helping health care workers to maintain safety during aerosolizing procedures like endotracheal intubation [13,14]. Infected physicians even chronicled their disease course on Twitter to educate followers in a novel way that would not have even been possible 15 years ago [14]. Similar to the global response to the Zika virus, physicians and public health organizations such as the US Centers for Disease Control and Prevention (CDC) and the World Health Organization also used Instagram to spread information to health care professionals and the general public from verifiable sources [15-17]. This rapid and efficient dissemination of information illustrates the significant influence social media can have on the spread of medical literature and knowledge among health care professionals.

The COVID-19 pandemic also disrupted medical education. It forced medical schools and residency and fellowship training programs to adapt to how they educate their trainees. Aided by virtual platforms such as Zoom and Microsoft Teams, formal educational lectures, noon conferences, grand rounds, and even medical conferences have migrated onto the web to adapt to the “new normal” [18]. With widespread cancellation of elective procedures, more procedural-based specialty training programs faced unique challenges to ensure their trainees would acquire adequate procedure skills. Gastroenterology fellowship programs adopted innovative virtual training webinars to strengthen participants’ theoretical background in endoscopy and video sessions to review common technical aspects of endoscopy; they also reinvigorated the use of simulation-based training, in which it has been shown that skills learned in virtual reality simulation-based training are transferable to real life [18-21]. Although Zoom and Microsoft Teams are the newest widely adopted virtual platforms for formal medical education, informal medical education has been present on multiple social media platforms for years. Moreover, with social distancing measures actively in place, social media platforms help provide health care professionals with opportunities to establish community and camaraderie that would otherwise not exist. Specific use case examples of educational opportunities on each social media platform are illustrated below.

### Facebook

The use of Facebook by patients to access and share medical information for chronic disease management has been well studied, and these studies may provide insight into how closed Facebook groups can be harnessed for medical education [7,22-30]. In some studies, researchers have looked at relatively small and homogenous groups of individuals who participate in well-moderated, closed Facebook groups to enhance weight loss in African American women [31], improve physical activity in patients with type 2 diabetes [30], and improve exercise motivation in patients with stable coronary artery disease undergoing cardiac rehabilitation [31]. These studies may provide important context on how Facebook groups can potentially enhance the learning experience of medical students. Although Facebook groups for medical education may pose privacy and logistical concerns, medical students are already using them to share learning tips, study strategies, and material and to discuss course content [32]. Faculty who engage in and moderate discussions with medical trainees in closed Facebook groups may help them better understand common problems and challenges that students encounter and, in doing so, may enhance the student experience [33]. 

### Twitter

The historically robust engagement of physicians with Twitter has led to several educational opportunities for medical trainees and attending physicians alike. Opportunities such as virtual case conferences, Twitter-based journal clubs, and “tweetorials” provide physicians with the ability to communicate with and learn from experts in their field whom they otherwise would not be able to access. For example, #MondayNightIBD is a weekly social media version of a multidisciplinary case conference. The weekly hashtag is used to identify discussion threads about the treatment or management of inflammatory bowel disease (IBD). It brings together clinicians from around the world to share their knowledge and research as it relates to a complex or controversial topic or situation [34]. These weekly discussions foster sharing of scientific data or guidelines when available, highlight areas where there is disagreement in data interpretation, and identify areas where more research is needed. These de facto case conferences also empower patients with IBD to help educate clinicians to better understand the patient experience and ultimately help improve patient care [35].

Twitter-based journal clubs are similar to contemporary journal clubs. They exist across various medical specialties, including but not limited to internal medicine, radiology, nephrology, urology, and echocardiography [36-40]. Typically, a chat is organized around a specific published article [37]. Participants use hashtags to follow subjects of interest and contribute to discussions [37]. Many journal clubs, such as #NephJC, involve live discussions over a specific time period that foster a conversational tone and instant communication. Other journal clubs, such as #UroJC, involve focused chats over a period of a few days to foster global discussion, which fosters participation when convenient for individual participants [36]. Twitter-based journal clubs promote global participation from individuals in different fields and institutions and provide participants with equal opportunity to participate in a timely and efficient manner [36]. Participants can engage directly with research authors, who may be able to provide nuanced insight that otherwise may not have been revealed, and simultaneously provide postpublication peer review [36-39]. Chan et al [41] outlined the steps to establish a web-based journal club, and although it is challenging to establish, promote, and maintain a Twitter-based journal club, it is comparatively easy to participate [41].

A tweetorial is a collection of threaded tweets with the goal of educating those who read them [42]. The impact of tweetorials is restricted only by the author’s audience. Users on Twitter can follow any number of individuals who use tweetorials as a teaching tool. Authors can use embedded pictures, videos, polls, or GIFs in tweets within the tweetorial thread, provide links to further reading or primary sources, and foster self-directed learning and teaching for health care professionals. Similar to Twitter-based journal clubs or case conferences, tweetorials enable individuals of varying hierarchical levels to directly interact who otherwise may not have the opportunity to do so [42]. Tweetorials can be used in formal medical education lectures and are a novel tool to summarize, educate, and disseminate complex topics in bite-sized teaching points.

### WhatsApp Group Chat

As the field of medicine grows, new ways also grow for health care professionals and those in training to digest educational material. In formal medical education classrooms, didactic lectures still predominate. Residency and fellowship training programs as well as continuing education for attending physicians are often at least partly driven by case-based learning through direct patient care. These important teaching points that physicians experience daily are often difficult to translate into formal lectures; however, widely available smartphones and software applications such as WhatsApp are disrupting and enhancing modern medical education.

WhatsApp is a secure, encrypted messaging software app that is restricted to mobile devices [43]. It enables physicians to securely share messages, links, documents, files, photographs, and videos in a timely manner and is an ideal smartphone app for modern medical education. It has been used to enhance and stimulate medical student education as an adjunct to formal classroom and problem-based learning [44-47]. The Duke University cardiovascular disease fellowship program successfully implemented a WhatsApp group chat to enhance the education of its fellows and continuing education of attending physician faculty [43].

Coleman and O’Connor’s scoping review [44] detailed a practical and learning framework for those interested in establishing successful WhatsApp educational group chats. Many successful educational group chats implemented a faculty “champion” or leader to focus discussions and facilitate learning objectives. Some group chats implemented a prespecified curriculum, while others used a continuous learning environment seeded by real life clinical cases [43-47]. This approach may be ideal for smaller groups, such as residency or fellowship house staff. However, it can also be limited by the relatively small size of the group, as group chats are reliant on individual member engagement. Ultimately, these studies have shown that WhatsApp educational group chats, if structured well, create safe spaces on the web for peer discussion and are applicable in multiple fields and educational levels.

### Instagram

The intuitive and interactive design and widespread use of Instagram create multiple teaching avenues for physician educators and learning opportunities for medical trainees. Sharing images to educate other health care professionals is not a new concept; however, the means and ease of doing so have changed. In 1992, the *New England Journal of Medicine* (*NEJM*) introduced *Images in Clinical Medicine* [48]. Today, *NEJM* continues to expose readers and Instagram followers to classic medical images and diagnoses to remind us of their clinical importance [49]. Although most users access Instagram for entertainment, a large number of physicians run medical Instagram accounts that enable users to learn in a unique and informal manner across multiple specialties, including but not limited to cardiothoracic anesthesiologist Dr Rishi Kumar (@RishiMD) [50], interventional cardiologist Dr Ali Haider (@YourHeartDoc) [51], cardiac electrophysiologist Dr Hafiza Khan (@Heart.Beat.Doctor) [52], interventional gastroenterologist Dr. Austin Chiang (@AustinChiangMD) [53], and pulmonary and critical care intensivist Dr. Cedric Rutland (@DrJRutland) [54]. Medical images and videos shared on Instagram give users access to virtual mini-case presentations that enable users to learn small pieces of information that they otherwise would not have been able to find or access.

Instagram is an ideal medium to share visually appealing teaching points, and it has been described in several specialties, including dermatology, plastic surgery, radiology, infectious disease, and cardiology [55-60]. Specialists such as interventional cardiologists can easily share a descriptive case, serial electrocardiograms, and noninvasive and invasive (catheterization) imaging studies to illustrate pearls of wisdom about the art of medicine that may not be found in formal curricula [60]. The static page of an account enables health care professionals to curate a feed of teaching points with accompanying photos, videos, and written descriptions. Instagram stories complement static posts by enabling followers to directly interact with posted text, photos, or videos in real time. This also instigates further in-depth discussion beyond a single post.

For prospective medical students, Instagram Stories may show them a glimpse into the medical field to supplement formal shadowing opportunities. For medical students and resident physicians, Instagram can similarly supplement formal rotations to gain insight into various fields or niche specialties that they would otherwise not be exposed to in their current rotations. Moreover, learners can transcend geography, time zones, and schedules to engage and learn from educators whom they otherwise may not have had the opportunity to interact with. Importantly, this informal setting may also allow trainees to voice questions they may not otherwise feel comfortable asking. For educators, the Instagram platform can be used in parallel to complement formal didactic lectures, share unique and interesting cases, and continue to provide teaching points even after the formal lecture is complete. 

### YouTube

Videos are an excellent medium to illustrate highly complex medical concepts. Signaling this potential, in 2006, *NEJM* established *Videos in Clinical Medicine* to offer peer-reviewed educational videos. These videos are created for medical trainees to help them better understand complex procedures and advanced physical examination maneuvers to ultimately improve patient care [61]. In fact, supplemental patient education videos published on YouTube have been shown to improve patient understanding of dual antiplatelet therapy after drug eluting stent placement [62].

YouTube is the single largest video-sharing platform on the internet and is the leading free web-based source of videos used by students and health care workers worldwide [63]. A study of 91 second-year medical students found that 98% used YouTube as a web-based information resource. When a YouTube channel was created for these same medical students to compound their understanding of gross anatomy, 86% of the students accessed the channel, and 92% of these individuals agreed or strongly agreed that the channel helped them learn anatomy [64]. YouTube is clearly an effective medical education tool to improve trainee understanding and integration of information across a molecular and clinical level [65].

Numerous medical YouTube channels already exist. Some individual physicians use their channels to teach the general public about various health issues, such as Dr Danielle Jones, an obstetrician/gynecologist who produces content on her channel at *Momma Doctor Jones* [66]*,* and Dr Mikhail Varshavski, a family medicine physician better known on his channel as *Doctor Mike* [67]. Organizations and medical societies also provide high-quality medical educational videos but also focus on medical knowledge for the general public. These include the CDC [68], the American Heart Association [69], and health care systems such as the Cleveland Clinic [70] and Mayo Clinic [71]. Other hospital networks, however, feature videos that are specific to graduate medical education. The Houston Methodist DeBakey CV Education channel [72] features free educational videos of didactic courses, hands-on learning, and procedures for cardiologists, cardiovascular surgeons, and vascular surgeons. Several companies also provide high-quality medical education content specifically for students at various levels of training. Companies such as *Osmosis* [73], *OnlineMedEd* [74]*,* and *Dr. Najeeb Lectures* [75] are among the most popular channels that provide free videos with expanded levels of content with paid subscriptions.

### Podcasts

Podcasts are ideal media for the delivery of medical education due to their relatively low cost, ease of access, and rapidity of distribution. Podcasts offer medical trainees the ability to learn at their own pace and can reinforce contemporary in-person lectures and can even foster more meaningful and engaging lectures. Podcasts are increasingly popular among medical trainees, with an increasingly more favorable perception over traditional books and journals [76]. The popularity of podcasts in medicine has grown alongside their success in the general public. In 2019, 139 active medical education podcasts existed across 19 different specialties; emergency medicine, internal medicine, and pediatrics were the specialties with the most active podcasts [77].

Podcasts can have varying structure and focus. One popular podcast, *The Curbsiders* [78], has over 271 individual episodes and covers a wide array of individual topics across medical specialties and subspecialties. By interviewing and discussing topics with experts from an array of medical disciplines, the *Curbsiders* podcast can provide a “deep dive” into the diagnosis, management, and treatment of various medical conditions. Therefore, listeners are able to gleam valuable insight into the minds of experts they otherwise would not have access to. Other formats include a review of recent literature publications or as a companion to formal journal publications. For example, *This Week in Cardiology* [79] is a weekly podcast that delivers a summary of noteworthy publications in the field of cardiology; meanwhile, *JACC Podcast* [80] is another free podcast recorded by Dr Valentin Fuster, the editor-in-chief of the *Journal of the American College of Cardiology*, that highlights the journal findings and provides a short summary of each manuscript.

It remains difficult to objectively assess the clinical utility of podcasts in medical education [81]. Although few studies have rigorously studied the efficacy of podcasts as teaching tools in medical education, their widespread use and adoption is evident [81,82]. In 2017, in a survey of 356 emergency medicine residents, it was found that 88.8% listened to a medical podcast at least once a month and that 72.2% reported that podcasts changed their clinical practice either “somewhat” or “very much” [82].

## Discussion

### Challenges and Pitfalls of Social Media Use by Health Care Professionals

First, we must acknowledge the prevalence and spread of misinformation on social media. This issue was present prior to the COVID-19 pandemic and is being exacerbated by it. Translating one’s credibility in the medical community is often difficult to replicate on social media. Similarly, accounts with large followings may not have verifiable credentials to provide medical education. For instance, an analysis of dermatological hashtag use on Instagram showed that only 5% of the top dermatology-related posts were created by board-certified dermatologists [55]. This finding indicates that although many physicians and health care professionals may in fact be on Instagram and using it appropriately, the majority of the most popular posts are created by individuals giving advice who are not qualified to do so. Without widely effective medical therapies to treat COVID-19, clear communication with the general public is our most effective medical treatment to date and underpins the importance of combating misinformation on social media [83]. Although medical journals may provide open access to health care professionals, this research is not accessible to the general public, who receive most information through social media channels [84]. This topic warrants further discussion and research; however, this is outside the scope of this review.

There are several limitations in our review. Formal medical education programs adapted enthusiastically to physical distancing requirements during the ongoing pandemic; however, the effectiveness of these virtual learning modalities has not been extensively studied. It remains unclear if social media or virtual learning modalities are applicable as a true substitute when in-person learning is limited. Similarly, it remains difficult to study the effectiveness of individual components of social media in medical education due to the multifactorial nature of medical education and the individual user variation of social media. However, the utility of various aspects of social media, including Instagram Stories, tweetorials, YouTube videos, and podcasts, is evident. Future studies should focus on guiding clinical educators on how to best use these platforms effectively and appropriately for their respective specialty. Even prior to the COVID-19 pandemic, an increasing number of health care professionals began engaging across social media platforms to provide informal medical education. However, the degree to which these web-based social media platforms will continue to be wielded for meaningful medical education following the eventual recovery from the pandemic is yet to be seen. Additionally, the trend toward the permeation of medical education across social media is apparent on platforms such as Reddit, TikTok, and Clubhouse; however, due to the limited availability of studies assessing educational content on these platforms, they were not included in our review.

For health care professionals, uniform training in proper use of social media is often insufficient. Many medical and educational institutions forbid active social media engagement by their trainees or provide vague guidelines on its use. As a result, unprofessional or perceived unprofessional behavior by health care professionals remains an ongoing issue. Organizations such as the Association for Healthcare Social Media and social media campaigns such as #VerifyHealthcare are concrete steps by health care organizations and individual professionals to combat this chronic issue [85,86]. However, larger institutional culture shifts and further formal studies are needed to evaluate how best to leverage social media to positively impact medical education.

Although these challenges are not new, they do complicate the already difficult task of using social media as an educational tool. As previously detailed, WhatsApp has been successfully integrated into formal medical school classes and informal cardiovascular disease fellow training [43-47]. YouTube channels and podcast series may be some of the most effective methods for educators to supplement trainee education. However, there may be challenges to formally incorporate these media and platforms such as Instagram, Twitter, or Facebook into formal medical education curricula. Therefore, these platforms remain supplemental resources for trainees, professionals, and patients alike. Future studies should examine how to best supplement contemporary medical education with each respective social media platform.

Studies should isolate differences between educating health care professionals in various stages of training. We surmise that there will be specialty-specific variations with regard to ideal platforms as well.

Future social media studies should implement process-evaluation strategies to ascertain which specific aspects of social media have the greatest impact. A conceptual framework was developed to aid future researchers in establishing studies on social media. This framework, known as the Therapeutic Affordances of Social Media (TASoMe), is grounded by the biopsychosocial model, or the interconnection between biology, psychology, and socioenvironmental factors [87]. TASoMe has been used to study social media use in brain cancer, endometriosis, and mental health [87,88]. It can aid researchers in systematically generating evidence-based research in a stepwise fashion and can be particularly useful for future studies on Facebook groups to educate trainees on chronic disease management [87].

It also remains difficult to quantify the academic impact of physician engagement on social media. As health care professionals engage on social media, they will gradually redirect their time from other responsibilities. Unfortunately, contemporary criteria used by academic institutions to evaluate individuals for academic promotions and tenure may not fully encompass the impact of social media posts or publications [89,90]. Expanded altmetrics for each social media platform can supplement contemporary metrics that aid in academic promotion or financial reimbursement in contract negotiations.

Lastly, contemporary studies on Facebook in medical education focus on perceived digital professionalism and likely reflect generational attitudes toward social media [91,92]. For better or worse, some residency program directors routinely survey public social media profiles of potential candidates, which directly influences residency match rank lists [91]. Teaching institutions must adapt to the changing web-based landscape and integrate realistic social media best practice guidelines into formal medical school, residency, and fellowship training program curricula to ensure that current and future generations of physicians are well equipped to use social media platforms meaningfully, responsibly, and professionally. 

### Conclusion

Social media platforms may come and go, and their engagement patterns may fluctuate; however, their impact on modern society is incalculable. The seeds of social media were enriched by separate yet intertwined technological advances that served as the building blocks of a communication revolution and spawned these integrative and seemingly inescapable social media platforms. In a time period that requires novel communication and teaching methods, social media can put the “social” back into physical distancing and medical education. The characteristics of each social media platform endow them with unique communication capabilities that have never before been seen in telecommunication history. Their use as educational tools must be approached with accelerated caution and monitored as they are implemented. Further studies are needed to better understand how health care professionals can most effectively use social media platforms as educational tools. Health care professionals can approach social media engagement in the same ethical manner that they would with patients in real life; however, health care institutions ultimately must enable their health care professionals to do this by enacting realistic social media policies. Institutions should appoint clinicians with strong social media experience to leadership roles to spearhead these generational and cultural changes. Ultimately, social media is expected to play a permanent role in influencing lay public and trainee knowledge. Clinicians and institutions must evolve to embrace and champion these platforms to preserve educational integrity and public trust.

## Abbreviations

CDC: US Centers for Disease Control and Prevention
IBD: inflammatory bowel disease
NEJM: New England Journal of Medicine
TASoMe: Therapeutic Affordances of Social Media
